# Effect of garlic genotypes (*Allium sativum* L.) on phenotype, growth, yield-related attributes, and nutritional quality at Bule Hora agro-ecology

**DOI:** 10.1016/j.heliyon.2023.e16317

**Published:** 2023-05-19

**Authors:** Gebre Garmame Galgaye

**Affiliations:** a Bule Hora University, Bule Hora, Ethiopia

**Keywords:** Garlic production, Genotype, Impact, Nutritional quality

## Abstract

Inappropriate variety use in different agroecology is one of the primary issues which significantly affect garlic phenotype, growth, yield, and nutritional quality. For this reason, a trial was done during the 2022 main season at the demonstration site of Bule Hora University, to see the impact of genotypes on phenotype, growth, yield-related attributes, and nutritional quality of garlic. The experiment was composed of four varieties namely Kuriftu, Holleta, Chafe, Tsedey 92, and one local cultivar. The trial was set up in a randomized complete block design replicated four times. The output showed that garlic varieties statistically (p < 0.05) affected all phenotype, growth, yield-related attributes, and nutritional qualities. Using a variety of Holleta and kuriftu minimize days of emergence by 9.82 and 10.33 days after planting and 75% physiological maturity by 23.6 and 25.90 days after planting, respectively, beyond variety Tsedey 92. The highest marketable bulb yield (8.21 t/ha, and 8.18 t/ha) was observed from Kuriftu and Holleta, respectively. While the lowest (4.39 t/ha) was from Tsedey 92. The highest Ash, energy, and carbohydrate were recorded from Kuriftu and Holleta, while the least from the Local cultivar. However, the highest moisture content, protein, and fat were observed from local cultivars, while the least from Kuriftu, and Holleta. In general, garlic varieties significantly affected all the phenotype, growth, bulb yield-related, and Nutritional quality. Therefore, it can be concluded that using the variety Kuriftu, and Holleta at Bule Hora agroecology is suggested to boost early germination, better performance, marketable bulb yield, and nutritional quality of garlic.

## 1 Introduction

Garlic (*Allium sativum* L.) is believed to be originated in central Asia so called Kazakhstan [1]. It has been food and medicine for a long time in India and China for a year above 5000, while before 2000 B.C. in Egypt [2]. Today, a lot of countries like Brazil, Korea, Kenya, Japan, Tanzania, and Italy including Ethiopia have been using garlic as a seasoning agent in many food preparation cultures, and modern medicine [3]. Besides, research finding revealed that it is rich in volatile compounds, used for flavor, and non-volatile compounds such as phenolic compounds (sapogenins, flavonoids, proteins, and saponins) amides, nitrogen oxides [4], minerals (Se, P, and K), vitamins (vitamins of B complex, and vitamin C), and antioxidants [5]. According to Ref. [6], garlic bulbs consist of moisture (62.38–68.33%), ash (1.16–1.87 g/100 g f.w.), carbohydrates (23.13–36.03%), fat (0.12–0.37 g/100 g f.w), protein (4.62–7.45 g/100 g f.w.), and energy (122.94–163.69 kcal/100 g f.w.). The nutritional characteristics of the different genotypes of commonly taken foods lead to the maintenance of food diversity and improve nutritional quality, particularly in mal-nutrition populations [7]. Consequently, garlic heals communicable diseases effectively [8]. Particularly, it has anti-bacterial [9,10], anti-cancer, *anti*-inflammantory, antihypercholestrolemic [10], anti-viral [10], and antioxidant [5], antifungal [11] activity than other Allium species due to its vinyldithiin [11], allicin [5], diallyl polysulfide [9], and ajoene [11].

Different genotypes have a real impact on the nutritional quality of garlic yield. The selection of genotypes based on the agroecology requirements, and customer desired quality (standards) should be a paramount clue towards the nutritional quality enhancement of the end-products [6]. Even though garlic is reproduced asexually by cloves in most of the globe by farmers, there is a significant difference in morpho-agronomic traits. This is due to the presence of various genotypes, cultivars in similar areas for an extended time, and the development of mutations [12]. [6] have revealed a significant difference in moisture, ash, carbohydrates, protein, and energy, not only among garlic cultivars belonging to varied agroecology but also between cultivars of the same agroecology.

According to Ref. [13], there was a significant difference in genetic diversity between various garlic landraces at specific agroecology, whereas differences in cultivars were common because of the variety and adoption of planting material between the different growing areas. In Ethiopia, even though the agro-ecology feature suite production of garlic, many abiotic and biotic determinants such as limitation of irrigable land, lack of high-yielding varieties, use of inappropriate fertilizer type or rate, disease, spacing or plant population, clove size, agroecology, irrigation, and institutional constraints, led to low garlic production [12]. Most probably, the lack of high-yielding varieties in a specific area is the major and primary factor affecting the production of garlic via phenotype, growth, yield, and nutritional parameters. Several scholars have conducted trials on the effect of garlic varieties on the plant pseudo height, length of leaf, bulb diameter, clove number per bulb, the weight of the bulb, and yield-related attributes of the plant [[14], [15], [16], [17], [18], [19], [20]].

Garlic grows and has been cultivated in a broad range of agroecology conditions [9]. While it grows very-well sub-tropical to temperate or high lands, in well-drained sandy, silty-clay soils, pH of 7, and excellent storage condition [10]. Garlic producers around highland areas cultivate garlic both for partial home self-consumption and mainly for income generation out to local and international markets [12]. Even though Bule Hora has an opportunity and potential of agro-climate conditions for garlic production, current garlic productivity in the area is below potential and has decreasing pattern [12,[21], [22], [23]]. Similarly, Bule Hora, is the largest garlic production opportunity area in the Guji Zone of southern Oromia, because of its agro-ecology feature, the ample presence of precipitation, and the good edaphic physicochemical properties. However, the Bule Hora area has its unique agroecology in which no garlic varieties are tested and not compared with locally cultivated ecotypes in terms of phenotype, growth, yield, yield-related, and nutritional quality. This study reveals information as genotypes and commonly used local cultivar significantly influences the growth and nutritional composition of garlic yield at the Bule Hora growing area [24]. Moreover, soil quality and fertility have a direct impact on nutrients amount in food crops, especially on medicinal plants. Mostly clues, such as temperature, sunshine, and rainfall, species, soil mineral composition, and bioavailability play a crucial role in the mineral uptake of plants, affecting the growth, development, and nutritional compositions [25]. Thus, garlic production tests at chosen areas could be used to stabilize the composition of compounds and, then, the nutritional quality of the end product [24]. There is less scientific information regarding the effect of garlic genotypes on phenotype, growth, yield, yield-related traits, and nutritional quality at Bule Hora agroecology, Therefore, the main objective of this study was to assess the effect of different garlic *(Allium sativum* L.) genotypes on phenotype, growth, yield, yield related, and nutritional quality at Bule Hora agroecology.

## 2 Methodology

### 2.1 Study area description

The study was conducted at the demonstration site, east Africa during the growing period of 2022. The site is geographically between a latitude of 5.58333, a Longitude of 38.25, 5° 34′ 60″ north, 38° 15′ 0″ East, and an altitude of 1836 m (6024 ft) with an Oceanic climate (Köppen climate classification: Cfb) (https://en.db-city.com/Ethiopia--Oromia--West -Zone--Bule-Hora). They are characterized by bimodal rainfall with 1250 mm and 600 mm of highest and lowest average rainfall, respectively [26]. It is found at 467 km away from Addis Ababa southeast direction of the main asphalt road towards Moyale. Total land area coverage is about 6021 square kilometers of natural and plantation forest covering 18,413ha and 1567ha, respectively [26]. The area is also characterized by good physical and chemical properties which are suitable for garlic production (Table 1).

**Table 1:** Experimental soil characteristics.

Soil particle	Clay silt
Bulk density	1.23 g/cm
Total porosity	53.21%
EC	0.0587 ms/cm
Field capacity of soil	30.81%
Wilting point (permanent)	20.85%
P^H^-H_2_O	6.98
C	1.93%
N	0.45%
Ca	2.12 meq/l
*P*	70.68 mg
K	0.32 meq/l
Na	1.01 meq/l
Mg	1.90 meq/l
CO^−^_3_^2^	0.20 meq/l

### 2.2 Planting material, experimental design, and treatments

Four improved and released varieties such as Kuriftu, Holleta, Tsedey 92, Chafe, and one Local cultivar (commonly used by the Bule Hora garlic producers, control) were selected based on their availability and adaptability. The varieties were obtained from Debrziet/Bishoftu Agricultural Research Center, Institute of Ethiopia Agriculture Research (DZARC/EIAR). The description of the varieties is stated in Table 2. The trial was set up as RCBD (Randomized Complete Block Design) arrangement, replicated four times, 20 plots (5 genotype by 4 replication equals to 20 number of observation or n, similar for all traits) as per reference [27].

**Table 2:** Garlic varieties used for the experiment.

Number	Genotype	Released Years	Breeder
1.	Kuriftu	2010	DzARC/EIAR
2.	Hollata	2000	DzARC/EIAR
3.	Tsedey 92	1999	DzARC/EIAR
4.	Chafe	1999	DzARC/EIAR
5.	Local cultivar	Not released	Collected from the Bule Hora area

Debrziet/Bishoftu Agricultural Research Center, Institute of Ethiopia Agriculture Research (DZARC/EIAR).

### 2.3 Cultural practices

After plowing, and well harrowing, the study area was leveled thereby 20 cm beds high were prepared. 1 m between blocks and 0.5 m between plots were used, respectively. 2 square meters of plot area were used. Five rows per plot with the distance between rows and plant was 20 cm × 10 cm were employed. Before planting, the bulb was separated into cloves and upright planting was employed [28]. Uniform planting was done by hand with proper topsoil covering. Uniform clove type had been planted in May 2022, at some stage in the wet season consistent with the standard planting of 10 plants per row. All good agricultural practices have been carried out in keeping with the suggestion for garlic as indicated by Ref. [29]. The metrology data is also characterized by good ecological properties which are suitable for garlic production (Table 3).

**Table 3:** Metrology data of the study area.

Year	Month	Variable		
		Temperature (⁰C)	Precipitation (mm)	Vapor pressure (Pa)
2022	January	19.30	22.20	8.90
2022	February	19.50	15.30	9.20
2022	March	20.20	219.60	11.20
2022	April	18.90	236.90	12.80
2022	May	18.80	232.90	15.70
2022	June	18.60	64.00	14.40
2022	July	17.50	92.30	13.40
2022	August	18.10	47.50	13.60
2022	September	18.80	121.00	13.60

### 2.4 Soil sample and analysis

After the experimental site was selected, the place to be sampled was taken based on the experimental total area. The zigzag method was used to sample the soil of the experimental site. The sampling was taken from an average depth of garlic root as described by Ref. [31]. One kilogram of mixed sub-samples of the experimental soil was sampled, after labeling, brought, and analyzed for physical and chemical properties. The textural class was done by the Boycouos hydrometric according to Ref. [32]. Ref. [33] procedure was followed to compute bulk density. The pH, carbon content, nitrogen, percent soil organic matter, electro-conductivity, and other physicochemical properties were measured according to Refs. [32,33], respectively.

### 2.5 Data collection method

Phonological data such as 50% emergence (days after planting) and 75% maturity time (days after planting); growth data like the height of the plant (cm), leave number per plant (count), length, and diameter of the leaf (cm); and yield traits such as length (cm), diameter (cm), and weight of dry bulb (q ha^−1^); cloves number per bulb (count/number); length of clove (cm); marketable (medium to large sized cloves, 2–3.5) (tha^−1^), and non-marketable cloves (very low to low sized, 1–1.99) (tha^−1^); and total garlic bulb (tha^−1^) has been measured from three representatively random sampling plants per row, three mid rows of each experimental unit (plot), nine plant per plot and total of 180 plants indicated by the [34].

### 2.6 Nutritional quality analysis

#### 2.6.1 Standards and reagents

According to 99.9% of Acetonitrile, 95% of *n*-hexane, and 99.8% of ethyl acetate were High-Pressure Liquid Chromatography (HPLC) grade from Fisher Scientific (Lisbon, Portugal) [35]. A purification system Millipore Direct-Q (TGI Pure Water Systems, Greenville, SC, USA) was used to obtain pure water. Fatty acids methyl esters standard mixture (standard 47885-U) sugars, and organic acids standards were used (St. Louis, MO, USA).

Before nutritional analysis, a bulb of garlic was peeled to get individual cloves, peeled, and cut into small sizes as slices. Nine bulbs were sampled from each plot and were exposed to −80 °C freezing conditions and dried by freezing before analysis. The dried samples from each plot were fined by pestle and mortar. The fined samples were stored and exposed to −20 °C freezing conditions until analysis. American Organization of Analytical Chemists International (AOAC International) procedures were employed to determine Ash, proteins, carbohydrates, and fat, which are standard analysis methods [21]. The macro-Kjeldahl method was applied to determine nitrogen content (N), explained by AOAC method 978.04 [22], and protein contents were calculated by N × 6.25. A Soxhlet apparatus and petroleum ether, using AOAC 920.85 procedure were employed to determine crude fat [22]. AOAC method 923.03 was employed to determine ash contents by incineration at 600 °C achieving a constant mass weight [22]. The carbohydrate was determined by difference, while energy was illustrated by the equation:

Energy (kcal) = 4 × (g protein) + 4 × (g carbohydrate) + 9 × (g fat).

### 2.7 Data analysis method

The data collected from Phonological data such as 50% emergence and 75% maturity time; growth data like the height of plant leave number per plant, length, and diameter of the leaf; and yield traits such as length, diameter, and dry weight of bulb; cloves number per bulb; length of clove; marketable and non-marketable cloves; and total garlic bulb was computed using ANOVA by SAS programming language version 9.2 and LSD was employed to indicate the variation among varieties in mean values with a probability level of 5% [35].

## 3 Result and discussion

### 3.1 Phenotype of garlic as influenced by varieties

The current study indicated that varieties were statistically (p < 0.01) varied for the investigated phonological traits such as today to 50% emergency and 75% physiological maturity, indicating the existence of genetic variations among the considered varieties (Table 4).

**Table 4:** Mean of Day to fifty % germination (DAP), 75% physiological maturity (DAP), height of Pseudo plant (cm), number of leaf (cm), length (cm) and diameter (cm) leaf of garlic varieties at Bule Hora Agro ecology.

Varieties	Day to 50% emergence	Days to 75% maturity	Pseudo Plant height	Leaf number/Plant	Leaf length	Leaf diameter
Holleta	8.51a	123.7a	50.43a	16.1a	32.31a	2.12a
Kuriftu	8ab	121.4a	50.3a	15.6a	31.64a	2.03a
Chafe	15b	130.2b	42.21c	8.2b	26b	1.34b
Tsedey 92	18.33c	147.3c	47.04b	5.6 c	24.7bc	1.12bc
Local Cultivar	18.01c	148.9c	40.35cd	5.5c	22.9c	0.81c
LSD	1.43	5.4	3.21	2.1	3.21	0.5
CV (%)	7.4	8	10.2	7.5	11	12
*P*-value	0.0031	0.041	0.01	0.002	0.003	0.003

The same letter indicates that the means stastically do not differ (p < 0.05).

The present study revealed that Kurftu (8 DAP, Days after Planting) and Holleta (8.51 DAP) were the earliest varieties, followed by variety Chafe (15 DAP), while Tsedey 92 (18.33 DAP) and local cultivar (18.01 DAP) was the latest variety. Furthermore, the study indicated that there was no significant difference between Kurftu and Holleta varieties. Similar to the present study [16], revealed that days to 50% emergency have been significantly influenced by varieties, indicating Kuriftu variety achieved 50% emergence at 12 DAP, whereas varieties Bishoftu Nech (10 DAP), and MM-98 (35 DAP) have been earliest and latest, respectively. Besides [15], indicated that a higher number of days to emerge has been observed by Tsedey 92 (15.55 DAP), whereas the local one has emerged earlier (10.5 DAP). Confirming the current study, reference [20] also reported that among the tested garlic accessions collected from different parts of Ethiopia, days to emergence have been highly influenced by garlic accessions, due to the presence of real genetic variability which is paramount important for future garlic varietal breeding. The variation of varieties in days to 50% emergency reported by different scholars might be due to the different agroecology and soil characteristics of the experimental area. For instance in the current study Kuriftu variety early achieved 50% emergence at 8 DAP, while 12 DAP under the [16] study. This might be because of an elevation of 2740 masl, maximum and minimum temperature from 18 to 35 °C, respectively, and the average rainfall of the study site is 766.9 mm varied from the current work experimental site agroecology. Furthermore, soil characteristics might contribute to the variation expressing genetic potential.

Garlic varieties' days of 75% physiological maturity were significantly varied (Table 4). The present study shows that Days to physiological maturity range from 121.4 to 148.9 DAP. Variety Kuriftu and Holleta attained 75% physiological maturity within a short (121.4 and 123.7 DAP) period. While Local cultivar and Tsedey 92 variety attained 75% physiological maturity within a longer (148.9 DAP and 147.3 DAP, respectively) time compared with other varieties considered in this experiment. Several researchers reported that the variety Tsedey 92 has taken a long time raged from 137 to 140.4 DAP to mature, indicating variability of garlic in maturity among the varieties because of their genetic differences [14,18]. Besides, In line with the current study, evidence from northern Ethiopia indicated that the cultivar Felegdaero and Kuriftu improved variety has attained physiological maturity early [15,16]. Oppositely [36], observed that Tsedey 92 variety has been observed as an earlier variety. This might be due to the variability of the environmental conditions in the different experimental areas that led to the expressed genetic code. However, stastical differences have been not indicated among varieties Holleta, Chafe, Tsedey 92 variety, and local for physiological maturity [14].

### 3.2 Growth traits (pseudo plant height, and leaf traits)

#### 3.2.1 Pseudo height of garlic plant (cm)

There was a statistical (p > 0.05) difference between varieties in the pseudo height of the garlic plant. The longest plant height was measured from variety Holleta (50.43 cm), and Kuriftu (50.3 cm), while the shortest pseudo stem height was recorded from the local cultivar (40.35 cm), and chafe (42.21 cm). However, there was no significant difference between the variety Holleta, and Kuriftu (Table 4). Previously, researchers reported that garlic pseudo-plant height might be significantly influenced by variety. In line with the current study [19], revealed that Holleta with a plant height of 57.43 cm long has been reported in the Angot area, northern Ethiopia. Besides [16], also revealed that garlic pseudo height has been significantly influenced due to different garlic varieties (p < 0.01), indicating the longest pseudo plant height has been observed at Kuriftu next to the variety commonly used by the community called local. 10.13039/100014337Furthermore, the non-significance (p < 0.05) difference observed in plant height among varieties Holleta, and Kuriftu has been supported by previous experiments [19]. Contradicting the current finding [14], indicated that the shortest plant height (42.37 cm) of garlic has been observed from Holleta. In addition, varieties Tsedey 92 and local varieties have been recorded to be the tallest (68.26 cm) and shortest (62.02 cm) in garlic pseudo plant height, respectively [18]. The contradicting result obtained might be due to the environmental characteristic of the locations contributed to plant height growth expression.

#### 3.2.2 Leaf traits [number of leaves per plant (count/number), length (cm) and diameter of leaf (cm)]

The present finding indicated that different garlic varieties significantly (p > 0.05) varied in leaf number. The highest leaf number was counted from the variety Holleta (16.1 leaves per plant) and kuriftu (15.6 leaves per plant) followed by the variety Chafe (8.2 leaves per plant). However, the minimum number of leaves was recorded from the Local cultivar (5.5 leaves per plant), and Tsedey 92 (5.6 leaves per plant) (Table 4). The variation observed in the number of leaves per plant indicated the presence of genetic variation among the varieties. A similar study reported variety Kuriftu has been observed as one of the higher leaf-producing varieties [17]. In line with the current report, a small leaf number of 4.75 leaf/plant has been indicated in the Rie variety [36]. The current study disagreed with the findings of [18] reporting that a higher leaf number (16.53) has been recorded from variety Tsedey 92. The differences revealed in the different experimental areas between similar varieties indicate different research setups, agroecology, and experimental considerations.

Length and diameter of garlic leaf were statistically (p > 0.05) affected due to varieties. The longest leaf length of 32.31 cm, and 31.64 cm; and the wider leaf diameter of 2.12 cm, and 2.03 cm were recorded from varieties Holleta and Kuriftu, respectively (Table 4). Whereas, the shortest leaf length of 22.9 cm, 26 cm, and 24.7 cm; and the narrowest leaf diameter of 0.81, 1.34, and 1.12 were measured from local cultivar, variety chafe, and Tsedey 92, respectively (Table 4). The previous report revealed that genotype-50/03 and “Genotype-7/2003″ have been recorded as a shorter length of 32.3 cm, while “G-32/2003″ has been found to have the widest leaf diameter of 1.65 cm, and the narrowest leaf diameter of 1.35 cm has been recorded from “G-21/2003" [20]. Contrarily to the present study, the highest leaf length of 30.74 cm and diameter of 1.525 cm have been also observed from Tsedey 92, while the shortest length of the leaf of 26.07 cm and diameter of 1.224 cm have been found from a local garlic variety [28]. Moreover, supporting the present finding as there was no statistical difference among varieties Holleta and Kuriftu [28], study didn't indicate significant variation in the length of garlic leaf between varieties. However, regarding the garlic diameter of the leaf, genotype-"161-2″ is the narrowest diameter of the leaf measuring 1.295 cm between considered garlic varieties.

### 3.3 Garlic yield traits

#### 3.3.1 Bulb length (cm), and diameter (cm) of the garlic plant

The bulb length and diameter of the garlic plant were statistically (p > 0.05) influenced due to varieties (Table 5). The longest length of the bulb 5.51 cm and the wider bulb diameter of 3.9 cm were recorded from the variety Kuriftu followed by the variety Holleta with a 4.98 cm bulb length and bulb diameter of 3.7 cm (Table 4). Whereas the shortest bulb length and diameter of 3.00, 3.21 cm; 2.29 cm, and 2.31 cm were measured from local cultivars, and varieties Tsedey 92. The present work indicated that significant genetic variation in bulb length and diameter was observed among all the varieties except local and Tsedey 92 considered in the experiment. This agrees with the previous study by Ref. [16] reported that Kuriftu has scored a high average bulb diameter when compared with varieties such as Tsedey 92 and MM-98. On the contrary [18], reported that Variety Tsedey 92 has been observed as the longest bulb length (4.29 cm), similar to that of the local variety (4.22 cm). Similarly, it has been indicated that Tsedey 92 (3.12 cm), followed by Chelenko I (2.746 cm), has been recorded as a long bulb diameter variety, whereas a short garlic bulb diameter (2.298 cm) has been observed from a local check [14]. Contradictions might be due to the different genetic characteristics interacting with agroecology and agronomic practices.

**Table 5:** Mean of length and diameter of bulb (cm), weight of bulb (q ha^−1^), and number of clove bulb^−1^ (count) of different varieties at Bule Hora Agro ecology.

Varieties	Length of bulb	Diameter of bulb	Average dry weight of bulb	Number of clove per bulb
Kuriftu	5.51a	3.9 a	43.92a	16.2b
Holleta	4.98b	3.7b	42.74b	16b
Chafe	4.12c	3.01c	41.91c	18.9a
Tsedey 92	3.21d	2.31 d	41.08d	20.2a
Local cultivar	3.00d	2.29 d	41.01d	20.4a
LSD	0.51	0.32	0.49	2.3
CV (%)	8	10	11.2	12
*P*-value	0.0021	0.011	0.0021	0.012

The same letter indicates that the means stastically do not differ (p < 0.05).

#### 3.3.2 Average dry bulb weight (q ha^−1^)

The mean bulb of the garlic plant was highly (p < 0.01) affected due to varieties (Table 5). It has been reported by different scholars that bulb weight is affected by variety. The highest mean bulb of garlic weight was weighted at Kuriftu (43.92 q ha^−1^), and Holleta (42.74 q ha^−1^), respectively (Table 5). While the minimum mean bulb of garlic weight has been observed from the local cultivar (41.01q/ha), and variety Tsedey 92 (41.08 q/ha). In terms of average garlic bulb yield, all the varieties considered in the experiment significantly varied. Similarly, it has been reported that Holleta has one of the highest bulb-weighted variety yields of “42.74 q ha^−1^″, followed by Tsedey 92 of “40.88 q ha^−1^″ at “Angot kebele”, where Chefe of “16.07 q ha^−1^″ has produced the low-yielding dry bulb [19]. Furthermore, In line with the present study from Northwestern Ethiopia [16], reported that Tseday 92 has the least bulb weight. However, the current finding was in disagreement with the previous study, indicating a significant high bulb weight has been observed from variety Tsedey 92, while a low bulb weight by variety Holleta [14,18,28]. Consequently, the superiority and inferiority of the variety compared to each other's under different setups of study might be due to the genetic potential and environmental factors expressing the trait of mean bulb yield to explore. Besides, the difference observed in the weight of garlic bulbs between varieties could be attributed to vigorous growth traits, which led to more photosynthetic processes, raised carbohydrate accumulation, and other physiological processes that resulted in more weight of garlic bulbs.

#### 3.3.3 Number of garlic cloves per bulb (count/number), and length of clove (cm)

According to Table 5, Table 6 presentations; the Number of garlic cloves per bulb and length of garlic cloves were statistically (p < 0.05) varied. The highest garlic clove numbers bulb-1 (20.4), (20.2), and (18.9) were counted from Local cultivars, Tsedey 92, and Chafe, respectively (Table 5). While the minimum garlic clove number bulbs-1 was counted from Kuriftu (16.2), and Holleta (16) varieties, respectively (Table 5). The current study shows that there was no significant difference among varieties Tsedey 92, Chafe, Kuriftu, and Holleta in clove number per bulb. While all the varieties considered in the current experiment were significantly varied in clove length (Table 6). The longest clove length was observed from the variety Kuriftu (4.31 cm) followed by Holleta (3.45 cm). Whereas, the shortest was recorded from the Local cultivar and variety Tsedey 92 of 1.01 cm (Table 6). In the present study the secret of Tsedey 92, and Chafe varieties variety having high cloves bulb-1 is because of their small-sized cloves. Agree with the present study [17], indicated that garlic clove number per bulb ranged from 10.80 to 18.95, showing a significant difference between the tested germplasms. In agreement with the current study, reference [16] revealed that the maximum clove number bulb-1 has been counted by variety Kuriftu (16.68), while Tseday 92 has been a variety with a significantly minimum clove bulb-1. The range in the current study revealed the presence of a diversity of varieties for clove numbers bulb-1 hypothesized and also there is a significant potential for breeding programs in the future [17]. On the other hand, several studies reported opposite findings due to their environmental condition and experimental setup. Accordingly, a study done elsewhere indicated that the highest clove number per bulb (23.74) has been observed from Tsedey 92 [18]. On the other hand, the highest average clove length of 1.54 cm has been observed from the variety Tsedey 92 [28]. Reference [14] reported the lowest clove number has been observed from the variety Holleta (8.36). However, found un-significant variation in the clove number bulb-1 among the tested materials [28].

**Table 6:** Mean of. Clove length (cm), Marketable clove (tha^−1^), Unmarketable clove (t/ha^−1^), and Total yield (tha^−1^) of different garlic varieties at Bule Hora Agro ecology.

Varieties	Clove length	Marketable yield	Unmarketable yield	Total bulb yield
Kuriftu	4.31a	8.21a	0.21 b	8.42a
Holleta	3.45 b	8.18a	0.22 b	8.40a
Chafe	2.12c	5.97 b	0.31 ab	6.28 b
Tsedey 92	1.01d	4.39c	0.54a	4.93c
Local cultivar	1.01d	4.37c	0.53a	4.92c
LSD	0.51	1.54	0.25	1.67
CV (%)	7.9	7.3	9	11
*P*-value	0.0021	0.031	0.02	0.032

The same letter indicates that the means statistically do not differ (p < 0.05).

#### 3.3.4 Marketable (high to medium-sized, 2–3.5 g) and non-marketable (very low-sized, 1–1.99 g) yields (t ha^−1^)

The present study reported that different varieties statistically (p < 0.05) varied in the marketable and unmarketable bulbs (Table 6). The maximum marketable bulb yield was harvested on variety Kuriftu, and Holleta at 8.21 t ha^−1^, and 8.18 t ha^−1^, respectively followed by variety chafe with the marketable bulb of 5.97 t/ha (Table 5). However, the minimum marketable bulb yield of 4.39 t ha^−1^ and 4.37 t ha^−1^ was harvested on variety Tsedey 92, and local cultivar (Table 6). The current study indicated that there was an insignificant difference in marketable bulb yield between varieties Kuriftu, then Holleta. However, the highest unmarketable yield of 0.53t ha^−1^, and 0.54 t ha^−1^ was observed on the local cultivar, and variety “Tsedey 92″, while the minimum was harvested from other varieties such as Kuriftu, and Holleta, and chafe of “0.21 t ha^−1^, 0.22 t ha^−1^, and 0.31tha^−1^″, respectively (Table 6). The study shows also that the variety with high marketable yield produced less unmarketable and vice versa. The present study disagreed with the report indicating, that the Tsedey 92 variety recorded the maximum marketable bulb of “8.05″ t ha^−1^ [18]. Besides [28], revealed that Tsedey 92 is the better yielding, the high marketable clove of “311.5 g/plot”, and the low non-marketable clove of “1.3 g/plot” from the local one and “genotype-99-2". Furthermore, the variety of Bisheftu Netch has been an attraction in terms of marketable clove yield in their study [28]. This may be due to the environmental variability leading to high yielders or fewer yielders in a different area. In general, clove and bulb yield, size, and other morphological traits have a direct proportion to marketable yield, customer satisfaction, and market need. When the marketable garlic bulb or clove size and other leaf traits increase, garlic bulb or clove yield increases, resulting in better customer satisfaction, and market needs, and vice versa.

#### 3.3.5 The yield of the total bulb (t ha^−1^)

There were significant (p < 0.05) different variations among varieties in the total bulb (Table 6). The maximum garlic bulb yield was recorded at variety Kuriftu, and Holleta of 8.42 t ha^−1^, and 8.40 t ha^−1^, respectively followed by variety chafe of 6.28 t ha^−1^ (Table 6). However, the minimum bulb yield of 4.93 t ha^−1^, and 4.92 t ha^−1^was harvested on variety Tsedey 92, and local cultivar without Statistical difference (Table 6). In line with the present study [19], reported that compared with other varieties, Adizemene local and Holleta varieties have been observed as having the highest yields in qt/ha at Ginaza kebele. While Tsedey 92 variety had low bulb yields of 10.06 q/ha [19]. Besides, in total bulb yield, scholars indicated that there is a wide range of variation for bulb yield (2003.00–7328.00 kg/ha) among germplasm [14,17]. On the other hand, a high yield of 8.45 t/ha has been reported from the variety Tsedey 92, whereas a low yield of 4.34 t/ha from the Holleta variety [29]. Furthermore, a high yield of “8.98 t ha^−1^″ has been harvested from the variety Tsedey 92, similar to the variety Bishoftu Netch yield of “8.81 t ha^−1^″ and the minimum total yield of “5.37 t ha^−1^″ from a commonly used by farmers, indicating that varieties released for other areas or has been adapted and high-yielding might not be suitable, giving high for different areas [23]. Besides, it might be due to the genetic code contributing to large-sized garlic bulbs and cloves containing high food material reserves, which led the garlic variety to produce a large garlic yield compared with small-sized garlic bulbs and cloves that have small food material.

### 3.4 Nutritional quality of garlic genotypes

**Fig. 1:**
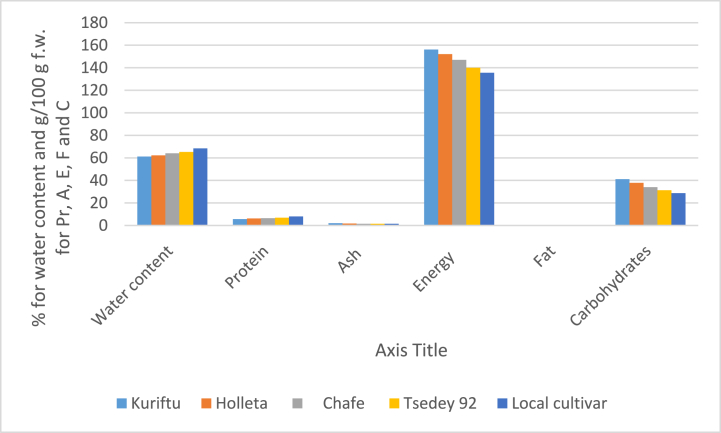
Mean of. Nutritional quality [water content in (%), Protein/Pr (g/100 g f.w), Ash/A (g/100 g f.w), Energy/E (kcal/100 g f.w.), Fat/F (g/100 g f.w), Carbohydrates/F (g/100 g f.w), and] of different varieties at Bule Hora Agro ecology.

In terms of nutritional quality, the present study reported that different varieties statistically (p < 0.05) varied in the water content, protein, ash, energy, fat, and carbohydrates at Bule Hora agroecology (Fig. 1). The value of nutritional qualities of the tested garlic varieties is within the range of the values revealed by Ref. [6] regarding water content, protein, ash, energy, fat, and carbohydrates. Besides [37], reported similar patterns regarding proteins, energy, fat, and carbohydrates. In line with the present study, different scholars revealed that there was a relation between agroecology, and genetic variation for different garlic cultivars, whereas differences in varieties were common because of genotype, and adoption of planting material among the specific growing condition. Specifically, nutritional traits such as water content, protein, ash, energy, fat, and carbohydrates influenced by genotype at Bule Hora agro-ecology are presented and discussed as follows; The maximum water content was determined by a local cultivar of 68.39%, followed by variety Tsedey 92 at 65.29%, and chafe of 64.08% (Fig. 1). However, the minimum water content of 61.19% and 62.28% was recorded from the variety Kuriftu, and Holleta (Fig. 1). The current study indicated that there was an insignificant difference in water content between varieties Kuriftu, and Holleta. This might be due to the genetic variability extent that existed among the tested genotypes and local cultivars in moisture content. Similarly [38], revealed that bulbs of dry garlic genotypes varied from 62% to 68% in water content.

The highest protein (7.97 g/100 g f.w) was detected from commonly cultivated, local cultivar, and, while the lowest was harvested from Kuriftu (5.65 g/100 g f.w) followed by Holleta (6.31 g/100 g f.w) as presented under Fig. 1. The study shows also that all the tested genotypes and local cultivars significantly varied in protein content. In line with this study [37], detected a significant protein of 4%–6% commonly in different cultivars. On the other hand, comparatively less content of proteins of 1.5%–2.1% was detected [38]. Moreover [39], indicated a significant variable among Chinese garlic and Spanish “Violetta” garlic. Furthermore, Spanish “Castano” garlic is blessed with 2.5 times more proteins than Spanish “Violetta.” Similar variability has been reported by Ref. [39] for Spanish “Morado” and Chinese garlic cultivars with high contents of *p*-hydroxybenzoic acid. On the other hand, similarity in other phenolic compounds has been reported in their experimental set-up for garlic cultivar analysis [39]. The variations in protein content observed by the current and previous genotypes study might be due to the genotypic variation, soil characteristics, and agroecology used for the experiment. Consequently, in the present, the soil characteristics, presented under Fig. 1, agro-ecology, and genotype and a local cultivar used for the experiment might be contributed to the high production of protein, one of the most important nutrients for human life.

The highest mean of garlic ash was recorded at Kuriftu (1.99 g/100 g f.w) followed by Holleta (1.75 g/100 g f.w), respectively (Fig. 1). While the lowest mean of garlic ash has been recorded from the local cultivar (1.51 g/100 g f.w), chafe (1.52 g/100 g f.w), and variety Tsedey 92 (1.56 g/100 g f.w). In terms of ash, there was no significant difference among the local cultivar, chafe, and Tsedey 92 varieties. Similar variability has been reported by Ref. [6] that ash in garlic genotypes varied from 1.16 to 1.87 g/100 g f.w, which is relatively near to the present study, indicating the extent of genetic variability among the varieties. Besides, results have been reported by Ref. [40], who reported insignificant differences determined in the content of ash.

The energy content of the garlic bulb was statistically (p > 0.05) influenced due to varieties at Bule Hora agroecology (Fig. 1). The highest energy with 156.19 kcal/100 g f.w was recorded from the variety Kuriftu (Fig. 1). Whereas the lowest energy content of 135.60 kcal/100 g f.w. Was measured from the local cultivar. The present work indicated that significant genetic variation in energy content was observed among all the genotypes and cultivars considered in the experiment. In line with the present study, the energy of the assessed garlic genotypes is indicated under Fig. 1 which is between the about of the values reported by Ref. [37]. Besides [6], revealed that significant variations were recorded among genotypes. Not only genotypes but also different growing areas influences the energy content of the garlic bulb.

There was also a statistical (p > 0.05) difference between varieties in the fat content of garlic bulbs (Fig. 1). The Local cultivar (0.49 g/100 g f.w) was the fattest garlic, while the low amount of fat was recorded from genotype Kuriftu (0.32 g/100 g f.w), followed by Holleta (0.39 g/100 g f.w.). Regarding carbohydrate content, the study shows an inverse proportion to fat content the highest carbohydrate was detected from Kuriftu (41.05 g/100 g f.w.), followed by Holleta (37.87 g/100 g f.w.). While the lowest was from the local cultivar 28.73 g/100 g f.w. Furthermore, this study revealed that all genotypes and cultivars were significantly varied in both fat, and carbohydrate (Fig. 1). The variations observed indicate that there might be the contribution of genetic information existing in the genotype and cultivar, as well as the agroecology and soil characteristics seems an expression of genes leads to determine the amount of fat and carbohydrate at Bule Hora area. In agreement with the present study, different findings revealed that there were significant variations between genotypes in fat, and carbohydrate [37,38,41]. Reference [38] revealed that 26%–30% range of carbohydrates from different genotypes of garlic. Furthermore [6], revealed that significant variation in carbohydrates (23.13–36.03%), and fat (0.12–0.37 g/100 g f.w) were recorded between genotypes, indicating genetic variation of the genotype, cultivars, and germplasms as well as growing area due to their geographical origin.

## 4 Conclusion

The main objective of the current study was to evaluate effect of phenotype, growth, yield-related attributes and nutritional quality of garlic genotypes, and local cultivar at Bule Hora agroecology. The experiment was conducted using completely randomized block design (RCBD) with four replication. Generally, the garlic genotypes and local cultivar tested revealed a significant (p > 0.05) variation in phenotype, growth, yield, and yield-related traits and nutritional quality at Bule Hora agroecology. This implies that different genotypes and local cultivars at Bule Hora condition shall have a paramount impact on phenotype, growth, yield, yield-related traits, and nutritional quality resulting in good quality of the pharmaceutical, and other medical sector input for their end garlic processed product. The presence of variability in the quality of nutrition is paramount for the production and productivity of garlic genotypes as a medicinal plant. Therefore, considerations might be devotedly attributed to select variety during production thereby boosting yield and productivity. Finally, Variety Kuriftu and Holleta can be recommended for production at Bule Hora agro ecology due to their advantageous phenotype, growth, high-yielding, and nutritional quality. Further study is also suggested for updating yield and revealing other nutrients like minerals, vitamins, specific proteins, carbohydrates, and other molecular characteristic of different garlic accession, germplasms, genotypes, and cultivar.

## Ethics approval and consent to participate

Not applicable.

## Data availability

Included in the main article.

## Declaration of competing interest

The authors declare that they have no known competing financial interests or personal relationships that could have appeared to influence the work reported in this paper.

## Figures and Tables

**Table:** 

Year	Month	Variable		
		Temperature (⁰C)	Precipitation (mm)	Vapor pressure (Pa)
**2022**	January	19.30	22.20	8.90
**2022**	February	19.50	15.30	9.20
**2022**	March	20.20	219.60	11.20
**2022**	April	18.90	236.90	12.80
**2022**	May	18.80	232.90	15.70
**2022**	June	18.60	64.00	14.40
**2022**	July	17.50	92.30	13.40
**2022**	August	18.10	47.50	13.60
**2022**	September	18.80	121.00	13.60
